# Tacrolimus-Associated Adverse Effects After Liver Transplantation: A Narrative Review

**DOI:** 10.3390/jcm15114176

**Published:** 2026-05-28

**Authors:** Nikola Bakovic, Ivana Pantic, Ivana Vasiljevic, Marko Vojnovic, Dusan Micic, Tamara Milovanovic

**Affiliations:** 1 Clinic for Gastroenterology and Hepatology, University Clinical Center of Serbia, 11000 Belgrade, Serbia; bakovicnikola27@gmail.com (N.B.); ilic.ivana04@gmail.com (I.P.); ivana.vasiljevic98@gmail.com (I.V.); marko.vojna@gmail.com (M.V.); 2 Faculty of Medicine, University of Belgrade, 11000 Belgrade, Serbia; ducamicic@gmail.com; 3 Clinic of Emergency Surgery, Emergency Center, University Clinical Center of Serbia, 11000 Belgrade, Serbia

**Keywords:** tacrolimus, adverse effects, liver transplantation

## Abstract

**Background:** Tacrolimus is currently the most commonly used immunosuppressive agent after liver transplantation, administered to prevent allograft rejection, but it is also associated with numerous adverse effects. **Purpose:** The aim of this review was to summarize the most clinically relevant and well-documented adverse effects of tacrolimus after liver transplantation, focusing on their mechanisms, clinical presentation, approximate incidence, and overall clinical significance. **Methods:** We searched the PubMed and Medline databases and included articles, randomized controlled trials, cohort studies, review articles, and case reports published between 2016 and 2026. **Results:** The most common causes of morbidity in the early days following liver transplantation and initiation of tacrolimus therapy are acute nephrotoxicity, systemic infections, and signs of neurotoxicity. In the majority of patients, these manifestations resolved after dose reduction or complete discontinuation of the drug. During the early post-transplant period, opportunistic infections were the main cause of morbidity, with bacteria being the most frequent pathogens, while mortality in certain fungal infections reached up to 50%. While acute toxicities are primarily influenced by drug dose, long-term use of tacrolimus, regardless of dose, can lead to the development of malignancies, metabolic disorders, cardiovascular diseases, and chronic kidney disease, with incidence estimates varying widely. **Conclusions:** Consistent monitoring of patients on tacrolimus is crucial to ensure maximum therapeutic benefit and to reduce the risk of associated adverse effects.

## 1. Introduction

Liver transplantation is the treatment of choice for patients with end-stage chronic liver disease and in cases of severe acute liver failure [1]. Allograft rejection remains one of the most important causes of morbidity and mortality following transplantation. It may occur as either acute or chronic rejection and is predominantly mediated by cellular immune mechanisms [2]. Over the past decade, tacrolimus has become the cornerstone of immunosuppressive therapy after liver transplantation [3]. Structurally, tacrolimus is a 23-membered macrolide lactone isolated as a product of the bacterium *Streptomyces tsukubaensis*. It was first described by Kino et al. in 1987 [4]. Previously known as FK506, tacrolimus binds to the FK506-binding protein (FKBP), thereby inhibiting the activity of the enzyme calcineurin phosphatase. This inhibition prevents the transcription of genes encoding interleukin-2 and interleukin-4, as well as nitric oxide (NO) synthesis, cellular degranulation, and apoptosis, ultimately blocking T-cell activation [3]. Although tacrolimus and cyclosporine share a similar mechanism of action, tacrolimus has largely replaced cyclosporine, another calcineurin inhibitor (CNI), due to its demonstrated superior efficacy and safety profile [5]. The introduction of tacrolimus has markedly reduced the incidence of acute allograft rejection from approximately 30–60% in the cyclosporine era to around 10–15% in contemporary practice, with a concomitant improvement in one-year graft survival to over 85–90% [6,7]. Tacrolimus is most commonly administered orally and is available in formulations with both immediate and prolonged release, which differ in their dosing schedules. Tacrolimus is a lipophilic compound metabolized in the liver and intestines by cytochrome P450 enzymes (CYP450), primarily the CYP3A subfamily. It is predominantly excreted via bile, with a reported half-life ranging from 4 to 41 h. Tacrolimus belongs to a group of drugs whose plasma concentrations require regular monitoring and maintenance within a narrow therapeutic range in order to prevent the occurrence of serious adverse effects [8]. This review focuses on the most clinically relevant and well-documented adverse effects of tacrolimus after liver transplantation, with emphasis on major toxicities rather than a comprehensive listing of all reported adverse events, and aims to present their underlying mechanisms, approximate incidence, clinical manifestations, and outcomes, with the ultimate goal of increasing clinicians’ awareness of tacrolimus-associated toxicity, promoting greater clinical vigilance, and highlighting the importance of further research in this field.

## 2. Materials and Methods

This study was designed as a narrative review. Therefore, a strictly predefined and reproducible literature search strategy was not applied. However, the PICO (Population, Intervention, Comparison, Outcome) framework was used as a conceptual tool to guide the literature search and structure the selection of relevant studies. The literature search was conducted between December 2025 and January 2026 using the PubMed and MEDLINE databases and included studies published between 2016 and 2026. The following keywords were used for the search: terms related to tacrolimus (“tacrolimus” and “FK506”) and liver transplantation (“liver transplantation” and “hepatic transplantation”), as well as terms describing adverse outcomes such as “adverse effects”, “safety”, “toxicity”, “nephrotoxicity”, “neurotoxicity”, “sepsis”, “infections”, and other relevant terms. The review primarily considered studies describing the use of tacrolimus after liver transplantation in adult patients, including both deceased and living donor transplantation, in which tacrolimus was administered either as monotherapy or in combination with other immunosuppressive agents. Studies of different designs, including randomized controlled trials, cohort studies, reviews, and case reports, were included to provide a comprehensive overview of the topic. Studies were initially screened based on titles and abstracts, followed by full-text assessment of potentially relevant articles. No formal quality assessment was performed due to the narrative nature of the review. However, the included studies were appraised with regard to their design, methodological limitations, and overall level of evidence. Greater emphasis was placed on higher level evidence when available, while case reports and small case series were primarily used to illustrate clinical presentations, and larger studies to provide an approximate estimation of incidence. Consequently, the findings should be interpreted in the context of inherent heterogeneity and differences in the level of evidence across the included studies.

## 3. Nephrotoxicity

One of the most common and clinically significant complications associated with tacrolimus therapy is nephrotoxicity. The use of tacrolimus after liver transplantation has been associated with both acute and chronic kidney injury [5]. Acute nephrotoxicity most commonly occurs shortly after the initiation of therapy. It is primarily mediated by activation of the renin–angiotensin system and reduced synthesis of nitric oxide, prostaglandin E2, and prostacyclin, ultimately leading to vasoconstriction of the afferent arteriole. This form of nephrotoxicity is dose-dependent [9]. A retrospective study which included 455 patients followed after liver transplantation demonstrated the highest incidence of renal impairment within the first three months after transplantation. The study also showed that patients receiving reduced doses of tacrolimus 6–10 ng/mL had significantly higher estimated glomerular filtration rate (eGFR) values compared with patients receiving standard or higher doses >10 ng/mL [10]. The dose-dependent nature of tacrolimus-induced nephrotoxicity was also confirmed by Nashan et al., whose study demonstrated lower mean eGFR values after 12 months of treatment with standard or high tacrolimus doses, compared with reduced tacrolimus doses used in combination with another immunosuppressive agent. The administration of everolimus in combination with reduced-dose tacrolimus—targeting trough concentrations of 3–6 ng/mL for everolimus and <5 ng/mL for tacrolimus—demonstrated a superior advantage in preserving renal function compared to standard tacrolimus regimens, with no significant differences in the incidence of allograft rejection [11]. A rare case of renal tubular acidosis associated with tacrolimus therapy has also been reported [12]. Chronic nephrotoxicity is associated with long-term, often years-long exposure to tacrolimus and is generally considered to be less clearly dose-dependent. The most significant drop in eGFR is seen in the first three months, up to 18 mL/min, with an additional decrease of up to 3 mL/min over the course of the first year. The average eGFR decline is more pronounced in individuals with pre-existing chronic kidney disease. Histopathologically, it is characterized by arteriolar hyalinosis, tubular atrophy and interstitial fibrosis, as well as glomerulosclerosis [9]. In two retrospective studies, chronic kidney disease was described as one of the most frequent complications associated with the use of standard tacrolimus doses, with reported incidences of 55.8% and 13% among the studied patients [13,14]. Analysis of data from the European Liver Transplant Registry (ELTR), including patients from 174 transplant centers across Europe, showed that kidney disease was reported as the cause of death in 4.3% of patients with fatal outcome following liver transplantation. Additionally, among deceased patients, the number of those using the immediate-release tacrolimus formulation was four times higher [6]. Of particular importance for clinicians is the potential for elevated serum tacrolimus concentrations and subsequent nephrotoxicity resulting from drug–drug interactions, primarily with agents that inhibit CYP3A4 activity. Therefore, cautious consideration is advised when prescribing concomitant medications [8].

## 4. Neurotoxicity

Tacrolimus-associated neurotoxicity after liver transplantation encompasses a broad spectrum of pathohistological changes and diverse clinical manifestations, ranging from tremor, speech disturbances, disorientation, and confusion to seizures and coma. One of the principal pathophysiological mechanisms involves increased nitric oxide production, disruption of the blood–brain barrier, and vasogenic edema of the white matter, as well as cellular cytotoxicity and apoptosis [15]. It is also important to distinguish tacrolimus-associated neurotoxicity from osmotic demyelination syndrome, which typically occurs shortly after transplantation and is associated with graft reperfusion, aggressive fluid resuscitation, cerebral hyperemia, and hyponatremia [16]. In the following section, we present eleven case reports published over the past 10 years describing various forms of probable tacrolimus-associated neurotoxicity after liver transplantation; these observations should be interpreted as descriptive signals of potential toxicity, rather than evidence of incidence or causality. The potential synergistic effects of concomitant immunosuppressants and patient-related factors must be carefully considered in clinical decision-making. Analysis indicates that the onset of symptoms may occur from several days to several months after the initiation of therapy. In most cases, signs of toxicity occurred despite therapeutic doses or serum concentrations of tacrolimus. The most common clinical presentation was altered mental status, followed by tremor and seizures. In the majority of cases, these manifestations were completely reversible and most often resolved after discontinuation of tacrolimus therapy (Table 1) [17,18,19,20,21,22]. A rare case of maculopathy has also been reported as an extracerebral manifestation of tacrolimus-related neurotoxicity [22]. Posterior reversible encephalopathy syndrome (PRES), which is characterized by an acute neurological disturbance with diverse symptomatology, represents an important manifestation of tacrolimus-associated neurotoxicity. Diagnosis is established by the presence of white matter lesions on T2-fluid-attenuated inversion recovery (FLAIR) magnetic resonance imaging (MRI). Both clinical symptoms and radiological findings typically resolve following the discontinuation of tacrolimus from the treatment regimen [20]. Furthermore, clinical attention should be directed toward the psychiatric manifestations of neurotoxicity, which can range from mild presentations to more severe conditions, such as hallucinations and paranoid disorder [19]. Previously, one of the potential strategies for addressing observed tacrolimus-induced neurotoxicity was conversion to cyclosporine. It was noted that this transition did not significantly increase the incidence of allograft rejection, while substantially reducing the probability of recurrent neurotoxic symptoms. Today, tacrolimus can be replaced by mTOR inhibitors or substituted with a combination of mycophenolate mofetil or mTOR inhibitors alongside significantly reduced tacrolimus doses [11,15,20].

## 5. New-Onset Diabetes Mellitus

Glucose metabolism disorder cannot be definitively attributed to a single factor, such as tacrolimus, as it represents a multifactorial condition. It has been established that dyslipidemia, overweight and obesity, and a pre-existing history of alcohol-related liver disease are significant factors contributing to the development of new-onset diabetes mellitus following liver transplantation. Nevertheless, tacrolimus exerts a significant impact through a distinct diabetogenic effect, primarily by inhibiting calcineurin phosphatase activity within pancreatic beta cells, thereby reducing insulin production. Additionally, tacrolimus may exert a direct cytotoxic effect on these cells [24,25]. The potential role of corticosteroids should also be considered, as they represent one of the most common causes of hyperglycemia following liver transplantation [25]. A review of two studies including a total of 686 patients reported an incidence of new-onset diabetes mellitus ranging from 21.5% to 24.8% within six months after liver transplantation. Furthermore, in addition to NODM, a significantly higher incidence of other metabolic conditions, such as overweight, obesity, and arterial hypertension, has been observed in patients treated with tacrolimus following liver transplantation, despite their clearly multifactorial etiology. Based on the calculated mean serum concentrations of tacrolimus, cut-off values were established above which the probability of developing these metabolic complications was significantly increased, and vice versa (Table 2) [25,26].

## 6. Post-Transplant Malignancies

The development of malignant tumors in patients after liver transplantation represents one of the most significant adverse effects associated with long-term immunosuppressive therapy, contributing substantially to morbidity and mortality [27]. However, this risk is multifactorial and influenced by additional patient-related factors. Several mechanisms have been proposed to explain tacrolimus-associated carcinogenesis. These include inhibition of T-cell activation, which reduces immune surveillance and the recognition of malignant cells, activation of the TGF-β signaling pathway, inhibition of DNA repair mechanisms, and stimulation of malignant cell proliferation through mTOR pathway activation. The most frequently reported tumors in this population include those associated with viral infections, such as Kaposi sarcoma, as well as genital and head and neck tumors. In addition, neoplasms occurring in areas with high environmental exposure, particularly skin cancers, are commonly described, along with tumors originating from the immune system itself, such as post-transplant lymphoproliferative disorder [27,28]. A large multicenter case–control study including liver transplant recipients treated with different immunosuppressive regimens demonstrated that prolonged exposure to tacrolimus was the only immunosuppressive exposure variable independently associated with cancer incidence in that analysis. This association was observed both for the development of de novo tumors and for the recurrence of hepatocellular carcinoma. Among 13,922 patients followed during a twelve-month observation period, malignant tumors were detected in 425 patients (3.05%). Compared with the control group, a statistically significant increase in cumulative tacrolimus exposure was observed after both three and twelve months, whereas combinations of other immunosuppressive drugs did not demonstrate such an association. Overall, these findings suggest that tacrolimus dose-reduction strategies may contribute to lowering the risk of carcinogenesis [28].

## 7. Opportunistic Infections

Immunosuppressive therapy, including tacrolimus, suppresses immune system activation and prevents graft rejection. However, through the same mechanism it increases susceptibility to infections. Infections represent one of the most common causes of morbidity and mortality after liver transplantation and may also contribute to graft dysfunction or rejection [29]. In general, the most frequent and severe infections after transplantation are bacterial in origin and typically occur shortly after the surgical procedure, most often in hospital settings and within the first month following transplantation [29,30]. Several additional factors may further contribute to the development of infections and worsen their clinical outcomes, including disruption of the mucocutaneous barrier and the presence of necrotic tissue, ischemia, and comorbid conditions such as diabetes mellitus, as well as other patient-related factors [29]. A large study published in 2019 analyzed data from the European Liver Transplant Registry (ELTR), including information from 174 transplant centers across Europe on the causes of death reported in 351 deceased patients who had received tacrolimus as the cornerstone of their immunosuppressive therapy. It should be noted that tacrolimus was considered as an isolated risk factor, although the concomitant use of other immunosuppressants may further increase patient susceptibility to infections. The results showed that infection was the leading cause of death, accounting for 107 cases (30.5%). Among patients in whom an infectious agent was identified, bacteria were the predominant cause of mortality, responsible for 43 deaths (12.3% of the total number of deceased patients). Fungal infections were identified as the cause of death in only two patients (0.5%). Furthermore, no statistically significant difference in overall infection-related mortality was established between patients receiving extended-release and immediate-release tacrolimus formulations. However, it should be noted that the interpretation of these findings is limited by an insufficient sample size [6]. Among fungal infections, invasive aspergillosis represents one of the most severe forms. Although its incidence is relatively low, approximately 1.8%, the associated mortality rate may reach up to 50% [31].

## 8. Sinusoidal Obstruction Syndrome

Sinusoidal obstruction syndrome (SOS) is a rare vascular liver disorder characterized by toxic injury to hepatic sinusoidal endothelial cells, inflammatory cell infiltration, and microthrombosis, collectively leading to sinusoidal obstruction, hepatomegaly, and portal hypertension. This condition was previously known as hepatic veno-occlusive disease (HVOD) until it was established that the primary vascular damage occurs at the sinusoidal level rather than in the hepatic veins. The gold standard for diagnosis remains liver biopsy [32]. SOS has particular relevance in liver transplant recipients. In a 2023 retrospective study, data from 331 liver transplant patients were analyzed, and SOS was identified in 3 patients (0.9%). The study also included a literature review, followed by the creation of a pooled cohort in which a total of 30 patients who developed SOS after liver transplantation were identified. Among them, 8 patients experienced an early, severe form of the disease (onset within 14 days post-transplant), while 22 patients developed a late-onset form (onset after 14 days post-transplant). Comparison of these two groups revealed significantly higher three-month post-transplant mortality in the early-onset group (86.1%) compared to the late-onset group (25.0%). Acute graft rejection was the most common cause of early-onset SOS, whereas immunosuppressive therapy was most frequently implicated in late-onset SOS [33]. Several case reports published within the past 10 years were identified where SOS was described in association with tacrolimus use after liver transplantation. Most patients presented with the late-onset form of the disease. For most patients, tacrolimus levels were within the recommended range at the time of clinical presentation (Table 3).

## 9. Discussion

Tacrolimus represents the cornerstone of immunosuppressive therapy following liver transplantation in many transplant centers worldwide, due to its demonstrated advantages over previously used immunosuppressive agents, particularly in preventing allograft rejection [8]. Achieving optimal dosing and maintaining therapeutic serum levels of tacrolimus remain major challenges due to the broad spectrum of adverse effects on one hand and the risk of graft rejection on the other. Tacrolimus dosing is guided by therapeutic drug monitoring based on trough concentrations, with higher target levels typically used in the early post-transplant period and gradual dose reduction during maintenance therapy, depending on clinical protocols [6,10,38]. This strategy allows individualized monitoring and dose adjustment according to patient-specific factors, including graft function, comorbidities, and concomitant medications. Pharmaceutical formulations with immediate-release and extended-release profiles have been developed [5]. Some studies demonstrated benefits from extended-release formulations, as they are associated with fewer adverse effects. The use of immediate-release formulations is associated with a higher risk of allograft rejection and patient mortality. Furthermore, a statistically significant improvement in three-year survival was observed in patients receiving extended-release formulations. However, further investigation is warranted [6]. Acute adverse effects are most often dose-dependent, while prolonged exposure can lead to gradual or chronic complications [9]. Since tacrolimus is primarily metabolized via the CYP3A4 pathway, it is essential to avoid medications that can inhibit or induce the activity of this enzyme. Various drug classes can increase serum tacrolimus concentrations through such mechanisms, thereby elevating the risk of adverse effects. The most potent inhibitory effects on CYP3A4 are exerted by voriconazole, posaconazole, macrolide antibiotics, and the antiviral agent letermovir. Conversely, moderate CYP3A4 inhibitors that also present significant interactions include fluconazole, clotrimazole, calcium channel blockers (such as diltiazem and verapamil), amiodarone, danazol, ethinyl estradiol, cimetidine, and proton pump inhibitors. On the other hand, agents that may decrease tacrolimus concentrations and thus increase the risk of allograft rejection include rifampin, rifabutin, phenytoin, carbamazepine, phenobarbital, and caspofungin.

On the other hand, CYP3A5 plays an important role in tacrolimus metabolism through genetic polymorphisms that affect enzyme expression, with individuals carrying the functional CYP3A5*1 allele exhibiting increased metabolic activity and faster drug clearance, and typically requiring approximately 1.5–2-fold higher tacrolimus doses to achieve target therapeutic concentrations compared to non-expressers [8,39].

In cases of tacrolimus-related toxicity or intolerance, alternative immunosuppressive agents such as cyclosporine, mycophenolate mofetil, and mTOR inhibitors (e.g., everolimus or sirolimus) may be considered. In clinical practice, tacrolimus is frequently used in combination with mycophenolate mofetil, while corticosteroid therapy remains an important component of immunosuppressive regimens, particularly in the early post-transplant period. Inhibitors of mTOR require careful therapeutic drug monitoring due to their narrow therapeutic window and potential for dose-related adverse effects. The choice of immunosuppressive strategy should be individualized based on patient-specific factors, including comorbidities, graft function, and clinical signs of drug toxicity [6,10,11,38]. Consequently, careful interpretation of adverse effects is required in patients receiving combination immunosuppressive therapy, as overlapping toxicities and drug interactions may complicate the attribution of clinical outcomes to tacrolimus alone.

Analysis of the available data indicates that adverse effects typically occur within a period ranging from a few days to several months or years after liver transplantation and initiation of therapy [17,18,19,20,21,22,23]. Nephrotoxicity is the most common and clinically significant adverse effect of tacrolimus, while morbidity and mortality are primarily influenced by the development of malignancies and severe systemic opportunistic infections due to the immunocompromised status of these patients [27,29]. Many tacrolimus-associated adverse effects are reversible, with clinical signs resolving after discontinuation of therapy, as seen in cases of acute kidney injury, neurotoxicity, and sinusoidal obstruction syndrome [10,15,32]. In cases of proven toxicity, it is necessary to reduce the dose of tacrolimus and combine it with other immunosuppressants—particularly when addressing mild neurological manifestations, such as muscle tremor, or early signs of renal impairment. Alternatively, in more severe cases, the drug should be completely discontinued and replaced with an agent from a different immunosuppressive class, such as mTOR inhibitors or cyclosporine [11,17,18,19,20,21,22,34,35,36,37]. This review highlights the most common and clinically significant adverse effects of tacrolimus in the past ten years, and is primarily focused on the selected clinically relevant and well-documented adverse effects of tacrolimus after liver transplantation. However, a range of complications should also be recognized, including dermatological reactions, such as acneiform eruptions, alopecia, and pruritus, gastrointestinal symptoms, electrolyte disturbances such as hyperkalemia and hypomagnesemia, hematological abnormalities including leukopenia and anemia, and cardiovascular effects such as arterial hypertension [8]. These effects are often multifactorial in origin, influenced not only by tacrolimus but also by concomitant immunosuppressive therapy, comorbidities, and the overall post-transplant clinical state, making it difficult to isolate the specific contribution of tacrolimus. Although generally less severe or less frequently reported in the recent literature, they remain clinically relevant and contribute to the overall toxicity profile in liver transplant recipients.

### Limitations

This study represents a narrative literature review; therefore, it did not employ strictly defined methodological procedures for data collection and analysis, as would be expected in a systematic review. Several limitations apply. First, only data on reported adverse effects of tacrolimus from studies published over the past ten years were considered, which inherently excludes a substantial body of relevant data from earlier publications. Additionally, the included studies were heterogeneous regarding study design, patient numbers, and the availability of relevant data on tacrolimus administration. This review provides an overview of tacrolimus-associated adverse effects and may serve as a practical resource in daily clinical practice, emphasizing the importance of close patient monitoring to promptly recognize potential adverse events related to this immunosuppressive therapy.

## 10. Conclusions

Tacrolimus remains the cornerstone of immunosuppressive therapy after liver transplantation. However, given the wide range of reported adverse effects, careful and regular monitoring of these patients is essential to ensure early detection and prevention of complications.

## Figures and Tables

**Table 1:** Tacrolimus-associated neurotoxicity.

Study	Study Type	Sample Size	Population Characteristics	Tacrolimus Dose/Concentration	Concomitant Immunosuppressive Therapy	Follow-Up Duration	Outcome Definition	Principal Findings
[17]	Case report	1	39-year-old male, liver transplantation for alcoholic liver cirrhosis	Dose: 10 mg/day, Concentration: 7.2 ng/mL	Mycophenolate mofetil	8 months	Neurotoxicity (talkativeness, tremor, and status epilepticus)	Complete resolution of symptoms occurred after tacrolimus was discontinued.
[18]	Case report	1	65-year-old male	NR	NR	NR	Neurotoxicity (rapidly progressive dementia)	Functional recovery was achieved after tacrolimus was discontinued.
[19]	Case report (case series)	1 (2)	54-year-old male, liver transplantation for alcoholic liver cirrhosis	Concentration: 8 ng/mL	Mycophenolate mofetil, prednisone	NR	Acute psychotic disorder	Complete resolution of symptoms occurred after tacrolimus was discontinued and replaced with cyclosporine.
[19]	Case report (case series)	1 (2)	57-year-old male, liver transplantation for alcoholic cirrhosis	Concentration: 12.7 ng/mL	Mycophenolate mofetil, prednisone	NR	Acute psychotic disorder	Complete resolution of symptoms occurred after tacrolimus was discontinued and replaced with cyclosporine.
[20]	Case report (case series)	1 (3)	60-year-old female, liver transplantation	Concentration: 10 ng/mL	Corticosteroids, mycophenolate mofetil	3 years	PRES (seizures and aphasia)	Complete resolution of symptoms occurred after tacrolimus was discontinued and replaced with sirolimus.
[20]	Case report (case series)	1 (3)	54-year-old female, liver transplantation for autoimmune hepatitis	Concentration: 8–10 ng/mL	Corticosteroids, mycophenolate mofetil	NR	PRES (seizures)	Complete resolution of symptoms occurred after tacrolimus was discontinued and replaced with sirolimus.
[20]	Case report (case series)	1 (3)	60-year-old female, liver transplantation, HCC	Concentration: 12.1 ng/mL	Corticosteroids, mycophenolate mofetil	NR	PRES (loss of consciousness)	Complete resolution of symptoms occurred after tacrolimus was discontinued and replaced with sirolimus.
[21]	Case report	1	51-year-old female, liver transplantation for PBC	Dose: 10 mg/day	Mycophenolate mofetil, corticosteroids	6 months	Parkinson’s and tremor	Complete resolution of symptoms occurred after tacrolimus was discontinued and replaced with sirolimus.
[22]	Case report (case series)	1 (2)	44-year-old male, liver transplantation	Dose: 4 mg/day Concentration: 3.94 ng/mL	NR	NR	Seizure	Resolution of symptoms following symptomatic therapy.
[22]	Case report (case series)	1 (2)	59-year-old male, liver transplantation	Dose: 3 mg/day, Concentration: 1.9 ng/mL	NR	8 months	Seizure	Resolution of symptoms following symptomatic therapy.
[23]	Case report	1	56-year-old male, liver transplantation for alcoholic cirrhosis	Concentration: 8.2 ng/mL	NR	6 months	Bilateral maculopathy and vision loss	Functional recovery was achieved after tacrolimus was replaced with cyclosporine and mycophenolate mofetil.

NR: Not reported.

**Table 2:** Incidence of new-onset diabetes mellitus associated with tacrolimus use and tacrolimus cut-off values.

Study	Study Type	Sample Size	Outcome (New-Onset Diabetes Mellitus)	Tacrolimus Cut-Off Values
[25]	Retrospective cohort study	528	131 (24.8%)	5.89 ng/mL
[26]	Retrospective cohort study	158	31 (19.62%)	5.9 ng/mL

**Table 3:** Review of cases of SOS associated with the use of tacrolimus.

Study	Study Type	Sampe Size	Population Characteristics	Tacrolimus Dose/Concentration	Concomitant Immunosuppressive Therapy	Follow-Up Duration	Outcome	Principal Findings
[34]	Case report (case series)	1 (3)	43-year-old male, liver transplantation for HBV cirrhosis	Concentration: 8.0 ng/mL	Mycophenolate mofetil,	NR	SOS diagnosis confirmed clinically and pathohistologically	Lethal, gastrointestinal hemorrhage
[34]	Case report (case series)	1 (3)	56-year-old male, liver transplantation for HBV related HCC	Concentration: >30 ng/mL	NR	NR	SOS diagnosis confirmed clinically and pathohistologically	Resolution of symptoms and pathohistology changes after discontinuation of tacrolimus
[34]	Case report (case series)	1 (3)	57-year-old male, liver transplantation for HBV related HCC	Concentration: 6.9 ng/mL	NR	NR	SOS diagnosis confirmed clinically and pathohistologically	Resolution of symptoms and pathohistology changes after discontinuation of tacrolimus
[35]	Case report	1	59-year-old male	Concentration: 7.7 μg/L	NR	NR	SOS diagnosis confirmed clinically and pathohistologically	Resolution of symptoms after switching to mycophenolate mofetil and cyclosporine
[36]	Case report	1	41-year-old male, liver transplantation for Mb. Wilson cirrhosis	Concentration: 6.1 ng/mL	Mycophenolate mofetil	NR	SOS diagnosis confirmed clinically and pathohistologically	Resolution of symptoms after switching to mycophenolate mofetil and cyclosporine
[37]	Case report	1	59-year-old male, liver transplantation for alcoholic liver cirrhosis	Concentration: 16.7 μg/L	Mycophenolate mofetil	6 months	SOS diagnosis confirmed clinically and pathohistologically	Resolution of symptoms after switching to mycophenolate mofetil and cyclosporine

NR: Not reported.

## Data Availability

No new data were created or analyzed in this study.
